# Improved DCT-Based Nonlocal Means Filter for MR Images Denoising

**DOI:** 10.1155/2012/232685

**Published:** 2012-03-07

**Authors:** Jinrong Hu, Yifei Pu, Xi Wu, Yi Zhang, Jiliu Zhou

**Affiliations:** ^1^College of Computer Science, Sichuan University, Chengdu 610064, China; ^2^College of Computer Science, Sichuan University, Chengdu 610065, China; ^3^College of Electronic and Information Engineering, Sichuan University, Chengdu 610064, China

## Abstract

The nonlocal means (NLM) filter has been proven to be an efficient feature-preserved denoising method and can be applied to remove noise in the magnetic resonance (MR) images. To suppress noise more efficiently, we present a novel NLM filter based on the discrete cosine transform (DCT). Instead of computing similarity weights using the gray level information directly, the proposed method calculates similarity weights in the DCT subspace of neighborhood. Due to promising characteristics of DCT, such as low data correlation and high energy compaction, the proposed filter is naturally endowed with more accurate estimation of weights thus enhances denoising effectively. The performance of the proposed filter is evaluated qualitatively and quantitatively together with two other NLM filters, namely, the original NLM filter and the unbiased NLM (UNLM) filter. Experimental results demonstrate that the proposed filter achieves better denoising performance in MRI compared to the others.

## 1. Introduction

Magnetic resonance imaging (MRI) is one of the most powerful imaging techniques [1] developed to study the structural features and the functional characteristics of the internal body parts. The visual quality of the MR images is normally corrupted by random noise from the acquisition process. Such a noise in MRI is mainly due to thermal noise that is induced by the movement of the charged particles in the radio frequency coils as well as the small anomalies in the preamplifiers.

Noise in MRI thus limits the visual inspection and the computer-aided analysis of these images. For example, it will introduce uncertainties in the measurement of quantitative parameters which hampers the estimation of the different properties of the analyzed tissues. Therefore, denoising should be performed to improve the image quality for more accurate diagnosis. Time averaging of the image sequences in parallel with acquisition is an effective acquisition-based noise filtering mechanism. However, this greatly increases the acquisition time and reduces the spatial resolution. Instead, filtering methods have been traditionally applied in the postprocessing stages. Such filtering methods have the drawback that, while removing noise, they may also remove high frequency signal components, thereby blurring the edges in the image and introducing some bias in the quantification process.

Several advanced image denoising methods can mitigate these drawbacks. For instance, anisotropic diffusion filters (ADFs) [2–4] are able to remove noise while respecting important image structures. In addition, more recently, wavelet-based filters have been applied successfully to MR image denoising [5–10]. Finally, a nonlocal means (NLM) filter, first introduced by Buades et al. [11, 12], has been recently improved and applied to MR data yielding the best results qualitatively and quantitatively when compared to other filtering techniques [13–19]. It is an efficient denoising method with the ability to result in proper restoration of the slowly varying signals in homogeneous tissue regions while strongly preserving the tissue boundaries.

However, the NLM filter may suffer from potential limitations since the calculation for similarity weights is performed in a full-space of neighborhood. Specifically, the accuracy of the similarity weights will be affected by the noise. What is worse is that many neighborhood pixels which are remarkably similar to the central pixel are also assigned slight weight. This would lead to bring side effect to the denoising results. For example, image's tissue regions may be weakened, especially small structural details and the distinct edge features.

Motivated by the above-mentioned problem, we integrate discrete cosine transform (DCT) into NLM filter to mitigate its limitation and propose a new filter. In the proposed filter, when performing the denoising, image patches are first transformed from time domain to frequency domain using DCT, and lower-dimensional frequency coefficients subspace of DCT is obtained through by Zigzag scan. Consequently, similarity weights are computed in this subspace with robustness to noise rather than the full space. Therefore, the accuracy of similarity weights is improved and more similar pixels can be obtained in the search window. Finally, considering the characteristics of Rician noise in MR image, the unbiased correcting is carried out to eliminate the biased deviation. The proposed filter has been compared with several methods presented recently, showing an improved performance both on vision and complexity.

The rest of this paper is organized as follows: Section 2 elaborates the proposed methodology and explains the materials and quality metrics used for validation. Then, the experimental results are presented in Section 3 and the discussion of experiments is presented in Section 4. Finally, the conclusion is presented in Section 5.

## 2. Materials and Methods

### 2.1. Nonlocal Means Algorithm

In the classical NLM algorithm [11], *u* is the discrete image with noise free, *n* is the noise and *v* is the noisy observation of *u* which is defined as *v*(*i*) = *u*(*i*) + *n*(*i*) at each pixel *i*. Let *N*_*i*_ denote an *r* × *r* square neighborhood centered on the *i*th pixel and *p*(*N*_*i*_) denote a matrix or a patch whose elements are gray level values of *v* at pixels in *N*_*i*_. We also define *S*_*i*_ as a square search-window centered on the *i*th pixel. An estimator for *u*(*i*), defined in the NLM algorithm [11], which is written as follows:

(1) $$\begin{array}{c} {\hat{u}}_{\text{ NLM}}\left(v\left(i\right)\right)=\underset{j\in S_{i}}{\sum }w\left(i,j\right)v\left(j\right), \end{array}$$

(2) $$\begin{array}{c} w\left(i,j\right)=\frac{1}{Z\left(i\right)}e^{-{||p(N_{i})-p(N_{j})||}^{2}/h^{2}}, \end{array}$$

where *Z*(*i*) = ∑_*j*∈*S*_*i*__ *e* ^−||*p*(*N*_*i*_)−*p*(*N*_*j*_)||^2^/*h*^2^^ represents a normalizing term, *w*(*i*, *j*) denotes the family of weights that are represented by the similarities between two pixels *i* and *j*, which satisfy the following two conditions 0 ≤ *w*(*i*, *j*) ≤ 1 and ∑_*j*∈*S*_*i*__ *w*(*i*, *j*) = 1. The similarity between two pixels *i* and *j* depends on the intensity gray level matrixes *p*(*N*_*i*_) and *p*(*N*_*j*_), which is measured by a decreasing function of the weighted Euclidean distance ||*p*(*N*_*i*_)−*p*(*N*_*j*_)||^2^. *h* denotes the smoothing kernel width parameter that controls the extent of averaging operations.

The success of NLM algorithm is attributed to the redundancy that is available in natural images. MR images are composed of plentiful repeated structure and averaging them will reduce the random noise, so the NLM filter is very suitable to be used as the denoising tool for reducing the noise in MR images. However, the NLM algorithm calculates similarity weights between pixels *i* and *j* by Euclidean distance in the whole neighborhood. The accuracy of similarity weights is inevitable vulnerable to noise, especially when the level of noise is strong. Therefore, the process to calculate the similarity weights in original NLM filter may lead to limited accuracy and bring a side effect to the denoised MR image. This would cause the image's tissue regions to be weakened, with respect to the edges, and fine structures.

### 2.2. Discrete Cosine Transform

Discrete cosine transform (DCT) has been introduced in 1974 by Ahmed et al. [20]. It is a widely used method for image compression, and it can also be used to reduce the dimensionality of image data. Ahmed et al. have proved that the DCT's performance is very close to that of PCA's when the data have reasonably large values of adjacent element correlation, especially for the image data [20, 21]. Additionally, the basis of DCT is fixed so that the computation of DCT is data independent and can be performed by simple matrix operation. The definition of the DCT is

(3) $$\begin{array}{cc} C\left(m,n\right)= & \alpha \left(m\right)\alpha \left(n\right)\overset{r-1\;}{\underset{x=0\;}{\sum }}\overset{r-1}{\underset{y=0}{\sum }}p\left(x,y\right)\cos\left[\frac{\pi \left(2x+1\right)m}{2r}\right]\\ & \;\;\;\;\;\;\times \cos\left[\frac{\pi \left(2y+1\right)n}{2r}\right], \end{array}$$

where *C*(*m*, *n*) represents the DCT's coefficients, *p*(*x*, *y*) represents that the image data will be performed by DCT, *r* is the width or length of *p*(*x*, *y*), *m*, *n* = 0,1, 2,…, *r* − 1 and *α*(*m*) is defined as

(4) $$\begin{array}{c} \alpha \left(m\right)=\{\begin{array}{cc} \sqrt{1/r} & \text{for}\begin{array}{c} \;m=0, \end{array}\\ \sqrt{2/r} & \text{for}\begin{array}{c} \;m\neq 0 \end{array}. \end{array} \end{array}$$

**Figure 1:**
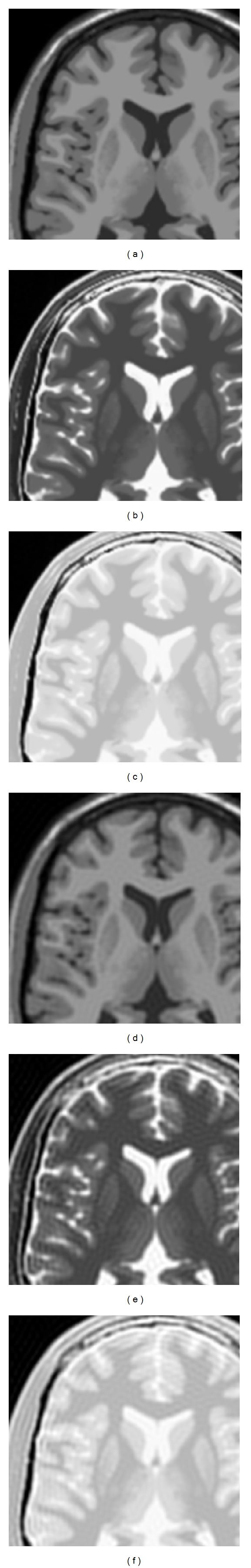
Energy compaction characteristics of DCT. The top row: images used before applying DCT; the bottom row: images reconstructed from only 25% coefficients.

**Figure 2:**
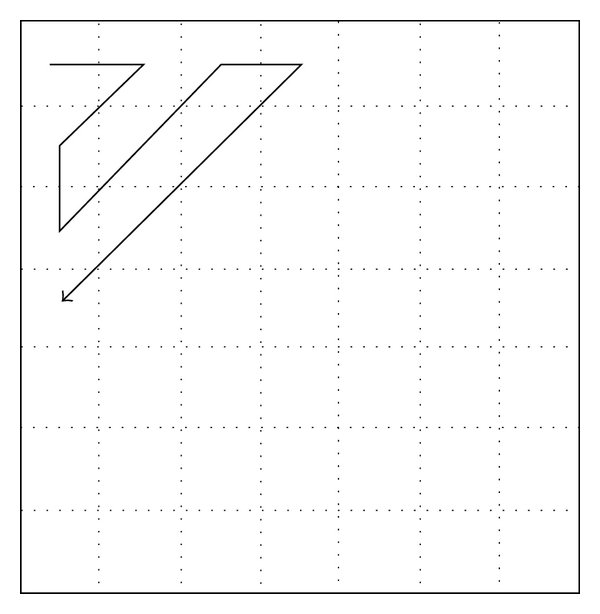
Zigzag scan used for selecting coefficients to compose subspace.

Due to DCT's promising characteristics, such as low data correlation and high energy compaction, it can be used as a very efficient method to decorrelate the image data [22]. In Figure 1, images in the top row are subregion of T1-weighted image, T2-weighted image, and PD-weighted image with size 128 × 128. Corresponding images in the bottom row are reconstructed from frequency domain with only 4095 coefficients. Note that the number of total coefficients is 16384: 128 × 128 and only 25% coefficients are used. In order to select suitable DCT coefficients to reconstruct an image, a Zigzag scan is performed by the way expressed in Figure 2. As a result, the image's sparse representation can be obtained through the DCT.

### 2.3. Discrete Cosine Transform-Based Nonlocal Means Algorithm

According to the above-mentioned content, we know that the DCT method has excellent energy compaction ability to pack input data into as few lower frequency coefficients as possible, thus it can be used as dimensional reduction technology to suppress the noise in image data [23, 24]. Motivated from the problems of traditional NLM method in Section 2.1, we present a new filter by integrating the DCT technology into NLM algorithm to boost the performance of MR image denoising.

In our method, we propose to replace the distances ||*p*(*N*_*i*_)−*p*(*N*_*j*_)||_*G*_*ρ*__^2^ in (2) by distances computed from the DCT subspace of *p*(*N*_*i*_) and *p*(*N*_*j*_). Let *M* be the number of pixels in the image neighborhood *N*_*i*_. Also let {*p*(*N*_*j*_)}_*j*=1_^*Q*^ be the set of all image neighborhood patches, where *Q* denotes the total number of pixels in the image. Firstly, image neighborhood blocks are transformed from time domain to frequency domain using DCT, and lower-dimensional frequency coefficients subspace of DCT is obtained through by Zigzag scan. This process is presented as follows:

(5) $$\begin{array}{cc} & C_{d}\left(N_{i}\right)\\ & =\{C\left(m,n\right)=\alpha \left(m\right)\alpha \left(n\right)\overset{r-1\;}{\underset{x=0\;}{\sum }}\overset{r-1}{\underset{y=0}{\sum }}p\left(N_{i}\right)\cos\left[\frac{\pi \left(2x+1\right)m}{2r}\right]\\ & {\;\;\;\;\;\;\;\;\;\;\;\;\times \cos\left[\frac{\pi \left(2y+1\right)n}{2r}\right]\}}_{\text{Zigzag}}, \end{array}$$

where *C*_*d*_(*N*_*i*_) represents the coefficients in DCT subspace of neighborhood *N*_*i*_, which are ordered by Zigzag scan sequence. Then we can derive

(6) $$\begin{array}{c} {||C_{d}\left(N_{i}\right)-C_{d}\left(N_{j}\right)||}^{2}=\overset{d}{\underset{k=1}{\sum }}{\left(C_{d}{\left(N_{i}\right)}_{k}-C_{d}{\left(N_{j}\right)}_{k}\right)}^{2}, \end{array}$$

where *C*_*d*_(*N*_*i*_)_*k*_ is the *k*th coefficient in *C*_*d*_(*N*_*i*_). Finally, we obtain the estimators with *d* ∈ [1, *M*] for our DCT-based filter and name it as **NLM-DCT** filter:

(7) $$\begin{array}{c} {\hat{u}}_{\text{NLM-DCT}}\left(v\left(i\right)\right)=\underset{j\in S_{i}}{\sum }w_{d}\left(i,j\right)v\left(j\right),\\ w{\left(i,j\right)}_{d}=\frac{1}{Z_{d}\left(i\right)}e^{-\sum_{k=1}^{d}{\left(C_{d}{\left(N_{i}\right)}_{k}-C_{d}{\left(N_{j}\right)}_{k}\right)}^{2}/h^{2}}, \end{array}$$

where *Z*_*d*_(*i*) = ∑_*j*∈*S*_*i*__ *e* ^−∑_*k*=1_^*d*^(*C*_*d*_(*N*_*i*_)_*k*_−*C*_*d*_(*N*_*j*_)_*k*_)^2^/*h*^2^^ is the normalizing term. Note that

(8)
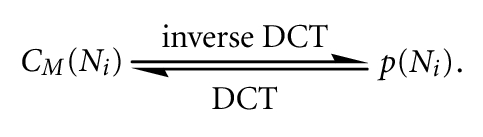


Therefore, the NLM-DCT filter with *d* = *M* is equivalent to the original NLM filter.

### 2.4. The Special Nature of the MR Images

The magnitude MR image is computed from the real image and imaginary image which contain Gaussian distributed noise, and the noise contained in the magnitude MR image follows a Rician distribution [25, 26]. The squared magnitude MR image (the value of each pixel in the image is the square of the value of the corresponding pixel in the original magnitude image) has a noise bias which is equal to 2*σ* ^2^ and is signal independent [7]. In concrete, for a given MR magnitude image *v*,

(9) $$\begin{array}{c} E\left(v^{2}\right)=u^{2}+2\sigma^{2}, \end{array}$$

where *u* represents the noise-free image of *v*; *v* ^2^ and *u* ^2^ represent the squared images of *v* and *u*, respectively.

To avoid such bias, Manjón et al. [15] and Wiest-Daesslé et al. [16] recently proposed a Rician-adapted version of the NLM filter. These two approaches are very similar. We used the correction scheme proposed by Manjón et al., which provides a slightly better correction of the imaged object in application. Manjón et al. [15] proposed to correct the unbiased intensity value as

(10) $$\begin{array}{c} {\hat{u}}_{\text{ UNLM}}\left(v\left(i\right)\right)=\sqrt{\max\left({\left({\hat{u}}_{\text{ NLM}}\left(v\left(i\right)\right)\right)}^{2}-2\sigma^{2},0\right)}, \end{array}$$

where ${\hat{u}}_{\text{NLM}}(v(i))$ denotes performing NLM denoising for pixel *i* of noisy image *v*.

Thus, our proposed filter with the correction scheme defined by Manjón et al. to perform unbiased denoising as (11) and name it as **UNLM-DCT** filter

(11) $$\begin{array}{c} {\hat{u}}_{\text{ UNLM}-\text{DCT}}\left(v\left(i\right)\right)=\sqrt{\max\left({\left({\hat{u}}_{\text{ NLM}-\text{DCT}}\left(v\left(i\right)\right)\right)}^{2}-2\sigma^{2},0\right)}. \end{array}$$

### 2.5. Materials

The well-known BrainWeb phantom [27–29] was used to evaluate the proposed approach in experiments. This database is widely used to test the performance of the denoising algorithms for MR images. Thus, it is convenient to compare the proposed filter with other denoising algorithms. In this paper, three images were simulated: T1-weighted MR image, T2-weighted MR image, and proton density-weighted (PD-weighted) MR image. They were simulated using SFLASH sequence, and each image contains 217 × 181 pixels. The performance of the denoising techniques is presented for these images with various Rician noise levels of the maximum of image intensity. The Rician noise was built from white Gaussian noise in the complex domain:

(12) $$\begin{array}{c} v_{r}\left(i\right)=u\left(i\right)+n_{1}\left(i\right),n_{1}\left(i\right)\sim N\left(0,\sigma \right),\\ v_{i}\left(i\right)=n_{2}\left(i\right),n_{2}\left(i\right)\sim N\left(0,\sigma \right), \end{array}$$

where *u* is the original image and *σ* is the standard deviation of the added white Gaussian noise. Finally, the noisy image is computed as follows:

(13) $$\begin{array}{c} v\left(i\right)=\sqrt{v_{r}{\left(i\right)}^{2}+v_{i}{\left(i\right)}^{2}}. \end{array}$$

**Figure 3:**
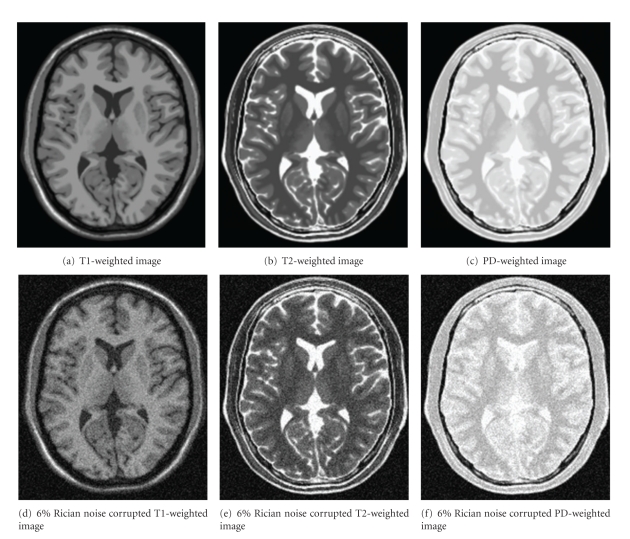
Samples of MR images with Rician noise. (a) to (c) are original images from the BrainWeb Database, (d)–(f) are noisy images of (a)–(c) with 6% Rician noise added.

Several levels of noise were added: 3%, 6%, 9%, 12%, 15%, and 18%. The first level (3%) represents the standard deviation of the added zero-mean white Gaussian noise, which is (3/100)*t*, where *t* is the value of the brightest tissue in the image. For T1-weighted image, T2-weighted image and PD-weighted image, *t* is 150, 250, and 255, respectively.  Figure 3 shows examples of the three images. The following experiments are all performed on these images.

### 2.6. Quality Measure

There are some criteria used to test the performance of the denoising methods. In the following, we will use three criteria to quantify the performance of each method: the peak signal noise ratio (PSNR), the residual image, and the visual evaluation.

The PSNR was computed as

(14) $$\begin{array}{c} \text{PSNR}=10\;\text{lo}{\text{g}}_{10}\frac{\text{Ma}{\text{x}}^{2}}{\text{MSE}}, \end{array}$$

where *Max*⁡ is the maximum of original image and noisy image, and MSE represents the mean square error estimated between the noise-free image and the denoised image:

(15) $$\begin{array}{c} \text{MSE}=\frac{1}{Q}\overset{Q}{\underset{i=1}{\sum }}{\left(u\left(i\right)-\hat{u}\left(i\right)\right)}^{2}, \end{array}$$

where *u*(*i*) and $\hat{u}(i)$ are the pixel values at position *i* of the original image and the denoised image, respectively. *Q* denotes the number of the pixels in each image.

The residual image is obtained by subtracting the denoised image from the noisy image [15]. It is required to verify the traces of anatomical information removed during denoising. Hence, this reveals the excessive smoothing and the blurring of small structural details contained in the image.

## 3. Results

### 3.1. Comparison of Weights Distribution

**Figure 4:**
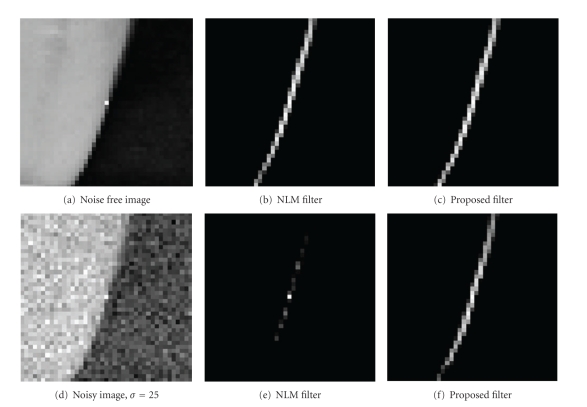
The comparison of the weights distribution used to estimate the central pixel of the left images with the noise-free and noisy conditions between the NLM algorithm and the proposed algorithm. (b) and (e) are the similarity weights distribution images for (a) and (d), respectively, which are calculated by the NLM filter in neighborhoods' full space of the central pixel. (c) and (f) are also the similarity weights distribution images for (a) and (d), correspondingly, which are obtained by the proposed filter in neighborhoods' DCT subspace of the central pixel. The weights distribution images are displayed with the logarithmic scale.

Figure 4 shows the comparison of weights distribution between the NLM filter and the NLM-DCT filter.  Figure 4(a) shows noise-free images; Figures 4(b) and 4(c) show the weights distribution of Figure 4(a) obtained by NLM and the UNLM. Additionally, Figure 4(d) shows the result of Figure 4(a) corrupted by noise with standard variance 25; Figures 4(e) and 4(f) display the weights distribution of Figure 4(d) produced by NLM and NLM-DCT, respectively. In this experiment, the parameters are assigned values as follows: the size of neighborhood patch is 7 × 7, the search window is the entire image with size of 41 × 41 and 10 coefficients selected by Zigzag scan were used to compose the lower DCT subspace of neighborhood.

### 3.2. Influence of the DCT Subspace Dimensionality

**Figure 5:**
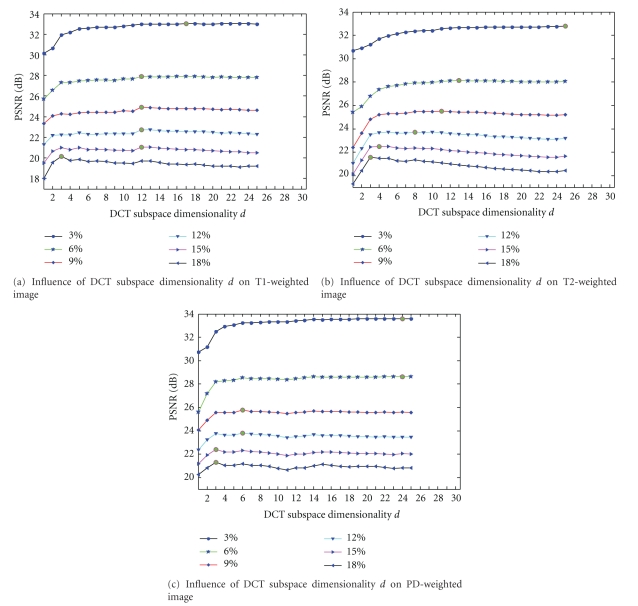
Influence of subspace's dimensional number *d* for the denoising effect. *S* is fixed to 11, *r* is fixed to 5, and *h* is set to get the best PSNR. (a) The experiments are performed on T1-weighted images; (b) the experiments are performed on T2-weighted image; (c) the experiments are performed on PD-weighted image.

Figure 5 illustrates the influence of the DCT subspace dimensionality parameter *d* for the denoising effect of the proposed filter under the condition of various Rician noise levels. Figures 5(a), 5(b), and 5(c) are the experimental results of the proposed filter on T1-weighted images, T2-weighted image, and the PD-weighted image, respectively. In this experiment, the sizes of search window and the neighborhood are 11 × 11 and 5 × 5 as same as the parameter setting in manuscripts [15, 19], which seems a reasonable value for medical images denoising. The value of parameter *h* is selected to obtain the best PSNR by exhaustion method.

In each graph, the PSNR of the denoised image is plotted against the DCT subspace dimensionality. The six curves are corresponding to the six input noise levels, which have a very characteristic shape around the optimal choice of *d*(*d*_opt_): steeply increasing PSNR for *d* < *d*_opt_, a knee around *d* = *d*_opt_ and flat or gradually declineing PSNR for *d* > *d*_opt_. The solid green circles in Figure 5 represent the curves' knee. 

### 3.3. Comparison with PSNR

Table 1 shows a comparison of the experimental results in PSNR. The data located at the “*Noisy*” row is the PSNR value of noisy images with the noise levels 3%, 6%, 9%, 12%, 15%, and 18%, respectively; the data lied in bracket “*()*” is the value of parameter *h* and *d*. For example, (3^4.38^) means *h* = 122.9677 and (3^4^, 17) expresses *h* = 81, *d* = 17. For all methods, the search window size is fixed to 11 × 11 and the neighborhood size is fixed to 5 × 5. The *h* is searched to obtain the best PSNR. For the proposed method, the subspace dimensionality *d* is set to *d*_opt_ corresponding to the best PSNR value in Figure 5.

**Figure 6:**
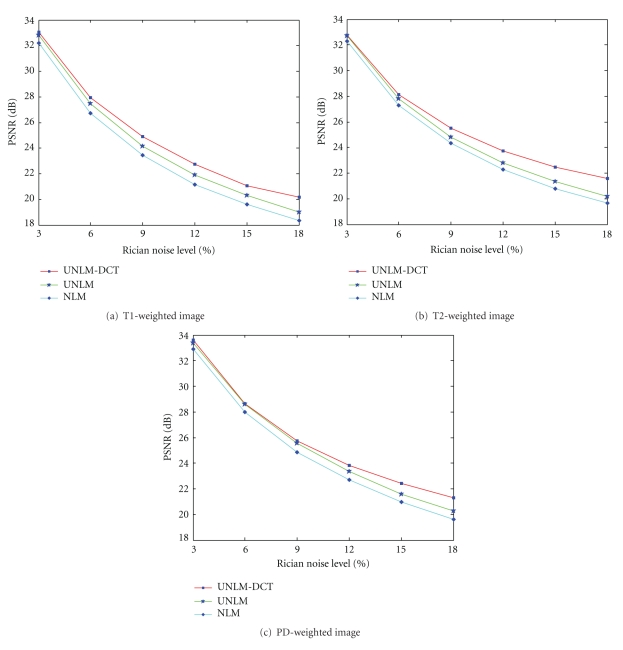
PSNR comparison of UNLM-DCT, UNLM, and NLM for different images and noise conditions. The proposed method outperforms the others in almost all the cases in terms of PSNR.

Figure 6 demonstrates the PSNR comparison of UNLM-DCT, UNLM, and NLM for different images and noise conditions more intuitively. Figures 6(a), 6(b), and 6(c) are show the PSNR value curves of the UNLM-DCT filter and other two filters for T1-weighted MR image, T2-weighted MR image, and PD-weighted image, correspondingly.

### 3.4. Comparison by Vision and Residual Image

**Figure 7:**
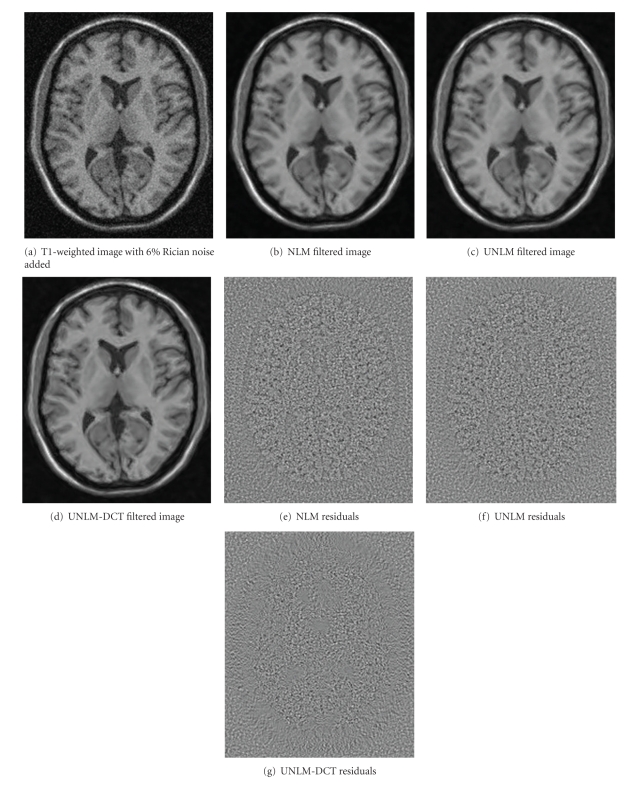
Qualitative comparison of experiment results on 6% Rician noise corrupted T1-weighted image. The quality of the proposed filter can be noticed in both filtered image and the corresponding residuals.

Figure 7 shows the qualitative comparison results of the three filters on T1-weighted image.  Figure 7(a) is the noisy image with 6% Rician noise added.  Figure 7(b) is the result image of the original NLM filter; Figure 7(c) is the result image of the UNLM filter; Figure 7(d) is the result of the UNLM-DCT filter. Figures 7(e), 7(f), and 7(g) are the residual images for NLM filter, UNLM filter, and UNLM-DCT filter, respectively, which were obtained by using the noisy image to subtract the filtered results.

**Figure 8:**
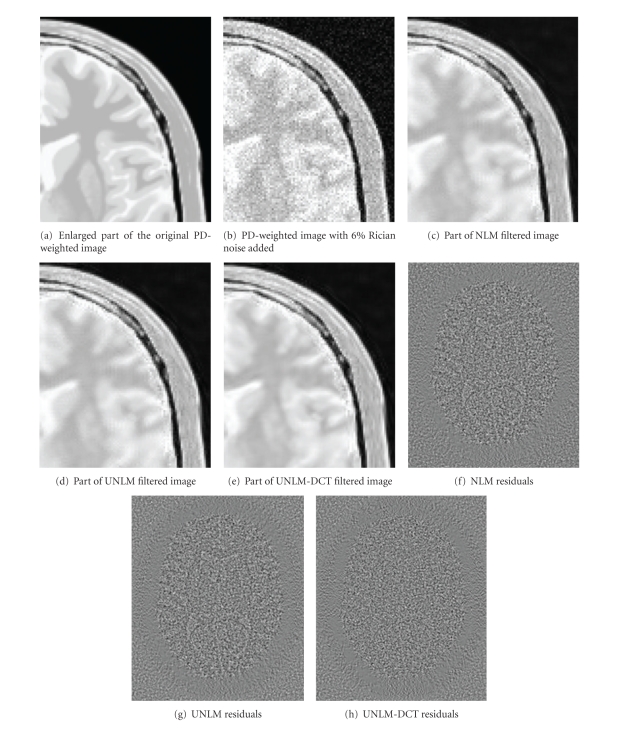
Qualitative comparison of experiment results on PD-weighted image. (a)–(e) are enlarged part of the experiment results with a factor of two.

Figure 8 shows the qualitative comparison results of the three filters on PD-weighted image with 6% Rician noise added.  Figure 8(a) displays an enlarged part of the original MR image; Figure 8(b) displays an enlarged part of the noisy image with 6% Rician noise added. Figures 8(c), 8(d), and 8(e) display the enlarged section of results obtained by NLM, UNLM, and UNLM-DCT from Figure 8(b), respectively. Figures 8(f), 8(g), and 8(h) show the residuals images of NLM, UNLM, and UNLM-DCT.

### 3.5. Comparison on Running Time

**Figure 9:**
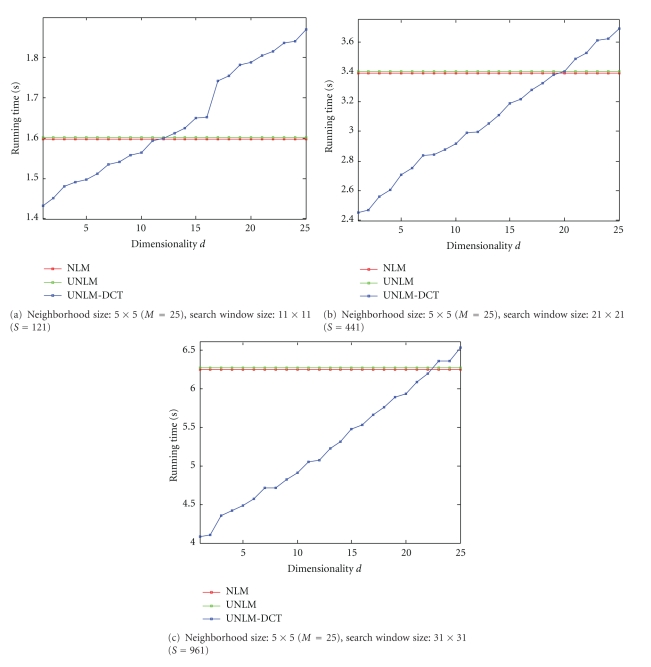
Computation times (in seconds) for the NLM filter, UNLM filter, and the UNLM-DCT filter. All methods were coded in MATLAB. The T1-weighted image is 181 × 217.

Figure 9 displays the computation times (in seconds) for the NLM filter, UNLM filter, and the UNLM-DCT filter with different search window sizes for the T1-weighted image. Figures 9(a), 9(b), and 9(c) show the filters' running time results with the neighborhood sizes of 5 × 5 and with the search window size of 11 × 11, 21 × 21, and 31 × 31, respectively. Those filters are implemented in MATLAB (Copyright The Mathworks, Inc.) on an Intel(R) Core (TM) i5-2400 CPU @ 3.01 GHz with 4G RAM.

### 3.6. Results on Real Data

**Figure 10:**
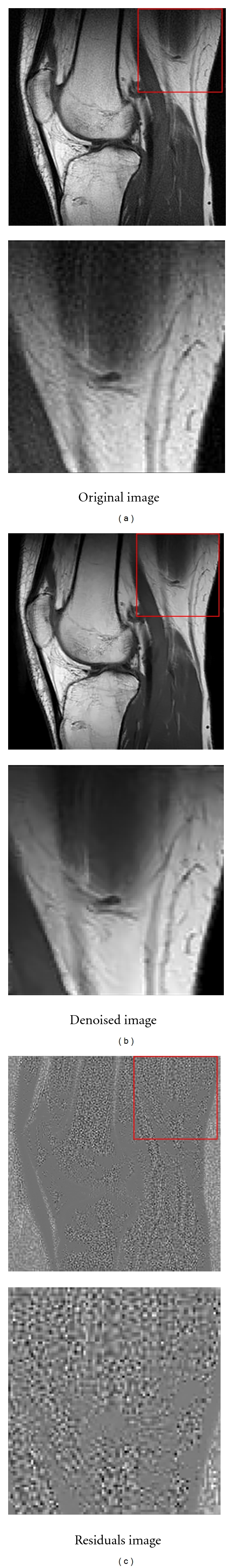
UNLM-DCT filter on a real T1-weighted sagittal MR image of the knee with an estimated noise standard deviation of 4.2. From left to right: original image, denoised image, and the difference image between them. Whole image is shown on top, and a detail of the rectangular region with red border is exposed on bottom.

To evaluate the proposed filter on real clinical data, we apply UNLM-DCT to a real T1-weighted sagittal MR image of the knee.  Figure 10 shows the results of the UNLM-DCT filter on this real knee MR image. The parameters are assigned as follows: the neighborhood size is 5 × 5, the search window size is 11 × 11, *h* = 4.2^2.1^, and *d* = 10. 

## 4. Discussion

The results show that the UNLM-DCT filter outperforms the NLM filter and UNLM filter among PSNR value, vision, residuals, and complexity. Therefore, we can infer that the UNLM-DCT has stronger denoising ability.

### 4.1. Comparison of Weights Distribution

The results shown in Figures 4(b) and 4(c) seem almost alike. Thus, we can see that the NLM filter and the NLM-DCT filter all can obtain the similarity weights accurately under the condition of no noise pollution. In addition, it can be easily observed from the result shown in Figure 4(e) that a mass of similar pixels would be lost in the case of the image polluted. This is because the similarity weights calculated in NLM filter are not too accurate. However, from the results shown in Figure 4(f), we can see that the NLM-DCT filter can still get more similar pixel under the noise condition.

### 4.2. Influence of the DCT Subspace Dimensionality

From these knees in Figure 5, we can see that the best *d* (*d*_opt_) ranges from 3 to 25 depend on the noise levels. For example, to the PD-weighted image, the best PSNR values were gained at *d*_opt_ = 24 with the noise level of 3% and 6%; *d*_opt_ = 6 with the noise level of 9% and 12%; *d*_opt_ = 3 with the noise level of 15% and 18%. In most cases, the best results are obtained at a relatively low DCT subspace dimensionality *d*_opt_, especially for higher noise levels. At the same time, the PSNR declines significantly beyond the knee whereas for lower noise levels it is flatter. In other words, the advantages of the proposed approach over the standard NLM algorithm increase with higher input noise levels. The increased accuracy at lower *d*_opt_ values can be attributed to the observation that distances computed in the lower dimensional space are likely to be more accurate than the full-dimensional space because DCT discards the most irrelevant dimensions. This explanation based on the accuracy of distances is also supported by the observation that the difference in PSNR among the proposed filter, the UNLM filter, and the NLM filter with increasing input noise level is shown in Figure 6.

In addition, we can see that the difference between the results for *d*_opt_ and *d* = 1 is around 1 to 3 dB in Figure 5. We can also know that the difference between UNLM-DCT and NLM seems to range from 0 to 2 db in Table 1. Therefore, we can infer that UNLM-DCT using only the zero DC component can provide results comparable to standard NLM, especially when the level of noise is strong (e.g., 12%, 15%, or 18%).

### 4.3. Comparison with PSNR

According to the PSNR data in Table 1, the UNLM filter and the UNLM-DCT filter achieve better results than the original NLM filter clearly. In addition, the UNLM-DCT filter performs better than the UNLM filter.

Furthermore, from the Figure 6 we can see that the UNLM-DCT outperforms the others for all test images with all noise levels in terms of PSNR. Specifically, the advantages of the UNLM-DCT increase with increasing noise level. When the noise is high (e.g., 12%, 15%, or 18%), UNLM-DCT significantly (almost greater than 1 dB difference) outperforms the UNLM and NLM. For low noise (e.g., 3%), UNLM-DCT performs slightly better than UNLM for all test images. Consequently, the distances in the DCT subspace become better approximations to the distances in the full-dimensional space. In other words, the difference between the two distance computations becomes minimal, which in turn results in very similar performance of the two approaches.

### 4.4. Comparison by Vision and Residual Image

From the Figure 7, we can see that the UNLM-DCT performs better than the NLM and the UNLM. Although the result obtained by the UNLM in Figure 7(c) is better than Figure 7(b) got by the original NLM, some regions were over smoothed and so that some useful information has been removed. On the other hand, from the residual images, we also can verify that the performance of UNLM-DCT is better than other two filters. Some structural details appeared in the residual images were gained by the NLM and the UNLM, which occur due to low accurate of similarity weights. Hence, on results of NLM filter and UNLM filter, the edge features are smoothened. However, the residual image obtained using UNLM-DCT does not show any traces of anatomical structures.

Figure 8 shows the results of three filters on PD-weighted image with 6% Rician noise added. Firstly, the comparison results on vision demonstrate that the UNLM-DCT can recover more anatomical information than NLM and UNLM. Secondly, the residual image produced by UNLM-DCT does not contain any structural details that occur due to oversmoothening. Hence, the UNLM-DCT can retain the distinct edge features while at the same time preserving small structural details.

However, there is still a little coarse on the edges in Figures 8(c), 8(d), and 8(e). As we all know, the quality of the NLM-based denoising result depends highly on the smooth parameter *h*, and a uniform optimum *h* is used to denoise the whole distorted image. The image contains low frequency regions, middle frequency regions, and high frequency regions. Therefore, there will be coarse edge effect in high frequency region when the smooth parameter *h* of weight function is small. Although a big value of *h* can eliminate the noise around the edges, lines, and other structure information regions, it makes the details oversmoothing in flat regions and middle frequency region. Thus, how to set the smooth parameter *h* adaptively and locally should be considered.

### 4.5. Comparison on Running Time

The computational complexity of NLM is *O*(|*Ω*| · |*S*| · *M*), where |*Ω*|, |*S*|, and *M* are the number of pixels in the image, in the search window *S*, and in the neighborhood patch *N*, respectively. In comparison, the complexity of UNLM-DCT has two components. One is the cost in using DCT for each pixels' neighborhood patch, which is *O*(|*Ω*| · *M* log^*M*^), and the other one is the cost in computing the similarity weights and the estimate for noisy pixel in a *d* dimensional DCT subspace, which is *O*(|*Ω*| · |*S*| · *d*). Therefore, the total complexity for UNLM-DCT is *O*(|*Ω*| · (|*S*| · *d* + *M* log^*M*^)). This should be smaller than the UNLM cost because typically |*S*| ≫ *M*.

From those results shown in Figure 9, we can see that those filters' running times are increased with the increase of search window size; and the computation cost of NLM and UNLM is almost the same. Moreover, the running time of UNLM-DCT is more than NLM and UNLM when *d* is closed to 25 because the UNLM-DCT contains a DCT for each neighborhood patch. However, the complexity of UNLM-DCT is less than NLM and UNLM under the condition of lower DCT subspace dimensionality. For example, the UNLM-DCT's running time is below the UNLM when *d* is smaller than 20 in Figure 9(b). Therefore, we can infer that if larger search windows *S* and larger neighborhood patches *N* are used, the computational savings over NLM and UNLM increase.

### 4.6. Results on Real Data

There is no ground truth used to select the filter's parameters when applying the UNLM-DCT to real clinical data. However, the quality of the denoising result depends highly on their setting, especially on the degree of filtering *h* and the DCT subspace dimensionality *d*. Thus, how to select the values for *h* and *d* automatically is a very significant issue in employing the UNLM-DCT filter to improve the real clinical MR images, which should be considered thoroughly in our future work. Now we assign the values to *h* and *d* based on the noise's standard deviation and the energy compaction property of DCT, respectively.  Figure 10 shows the results of the UNLM-DCT filter on a real T1-weighted sagittal MR image of the knee. In this experiment, *h* = 4.2^2.1^, which is assigned according to the estimated noise standard deviation of the knee MR image (the estimated noise standard deviation is 4.2, and it is calculated from the background of the squared magnitude knee MR image by Nowak's method [7]). With respect to the DCT subspace dimensionality, the PSNR curves shown in Figure 5 demonstrate that the optimal choice of *d* (*d*_opt_) is relative to the noise's level. They also indicate that the results of UNLM-DCT are not worse than NLM and UNLM for some chosen *d*. Therefore, we recommend selecting the value of *d* adaptively according to the energy compaction property of DCT (it is shown in Figure 1) and the estimated noise standard deviation of real MR data. For example, we use the Zigzag scan to select only 25% low frequency coefficients to compose the DCT subspace: *d* = (5 × 5) × 0.25 = 10. In addition, the neighborhood size and the search window size still are 5 × 5 and 11 × 11, respectively.

 The results shown in Figure 10 demonstrate that almost no anatomical information can be noticed in the residuals image and no artifacts are introduced in the denoised image.

## 5. Conclusion

This paper presents an improved NLM filter with preprocessing for MR images. Validation was performed on the BrainWeb dataset [27–29] and a real T1-weighted knee MR image, which showed an improved performance for different image types and levels of noise.

The contributions of the proposed filter mainly include calculating similarity weights in DCT subspace to reduce the disturbance of the noise for more accurate computation of the similarity and for much less computation complexity than the original NLM filter. Comparative experiments were performed on simulated MR image from the BrainWeb dataset and a real knee MR image to compare and analyze the proposed filter with the original NLM filter and the UNLM filter. Moreover, experimental results demonstrated that, by using the proposed UNLM-DCT filter, the noise bias can be corrected and the original information can be successfully restored.

In conclusion, the obtained results suggest that the application of the proposed filter may benefit many quantitative techniques that rely on good quality of the data. In this sense, applications such as segmentation, tractography, or relaxometry may take advantage from the enhanced data produced after the application of the proposed filter.

## Figures and Tables

**Table 1:** Comparisons of experimental results in PSNR.

Test images	Algorithms	Noise level (dB)					
		3%	6%	9%	12%	15%	18%
T1-weighted MR image	*Noisy*	29.86	24.3	21.26	19.56	18.35	17.44
	NLM *(h) *	32.21 (3^4.38^)	26.73 (6^2.94^)	23.44 (9^2.46^)	21.14 (12^2.24^)	19.61 (15^2.12^)	18.36 (18^2.04^)
	UNLM *(h) *	32.8 (3^4.38^)	27.46 (6^2.94^)	24.14 (9^2.46^)	21.9 (12^2.3^)	20.3 (15^2.12^)	19.02 (18^2.06^)
	UNLM-DCT *(h,d) *	33.02 (3^4^,17)	27.92(6^2.48^,12)	24.9 (9^2.08^,12)	22.76 (12^1.86^,12)	21.05 (15^1.74^,12)	20.16 (18^1.26^,3)

T2-weighted MR image	*Noisy*	30.48	25.21	22.44	20.66	19.4	18.45
	NLM* (h) *	32.3 (3^4.62^)	27.3 (6^3.02^)	24.32 (9^2.54^)	22.27 (12^2.3^)	20.8 (15^2.18^)	19.68 (18^2.06^)
	UNLM *(h) *	32.7 (3^4.62^)	27.79 (6^3.02^)	24.83 (9^2.62^)	22.8 (12^2.36^)	21.36 (15^2.18^)	20.19 (18^2.06^)
	UNLM-DCT *(h,d) *	32.77 (3^4.32^,25)	28.13 (6^2.64^,13)	25.53 (9^2.16^,11)	23.73 (12^1.86^,8)	22.49 (15^1.56^,4)	21.56 (18^1.44^,3)

PD-weighted MR image	*Noisy*	30.52	25.23	22.43	20.61	19.31	18.33
	NLM *(h) *	32.9 (3^4.54^)	27.98 (6^3.02^)	24.87 (9^2.54^)	22.7 (12^2.36^)	20.97 (15^2.18^)	19.63 (18^2.12^)
	UNLM *(h) *	33.36 (3^4.54^)	28.59 (6^3.02^)	25.55 (9^2.62^)	23.37 (12^2.36^)	21.57 (15^2.24^)	20.29 (18^2.12^)
	UNLM-DCT *(h,d) *	33.58 (3^4.16^, 24)	28.64 (6^2.72^, 24)	25.76 (9^1.92^, 6)	23.81 (12^1.74^, 6)	22.41 (15^1.44^, 3)	21.31 (18^1.32^, 3)
